# Supplementary figures and images for: The influence of truncating the carboxy-terminal amino acid residues of streptococcal enolase on its ability to interact with canine plasminogen
Source: PLoS One. 2019 Jan 17;14(1):e0206338. doi: 10.1371/journal.pone.0206338 (PMC6336276; doi:10.1371/journal.pone.0206338)

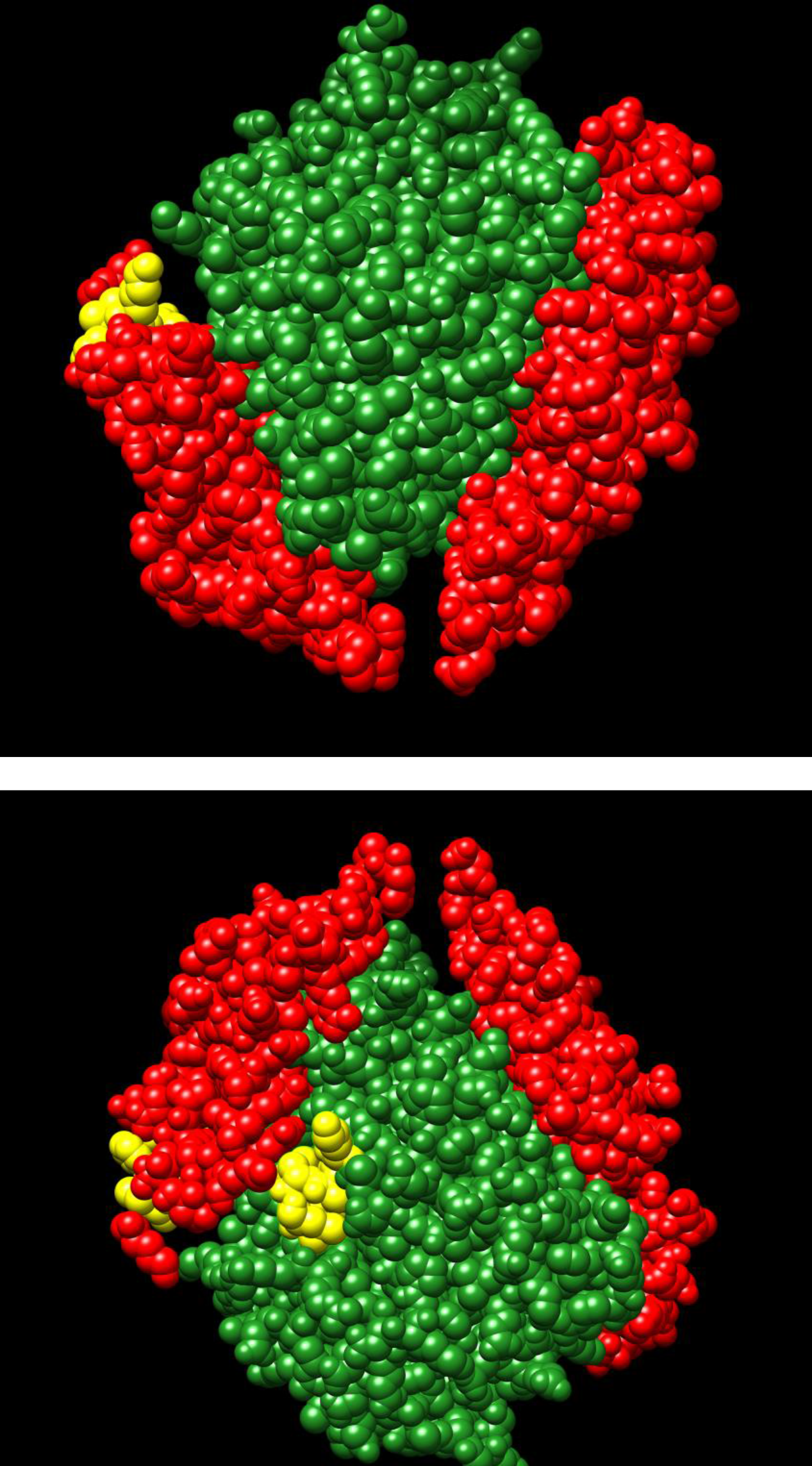

Supplement: S1 Fig — The illustrations are of the two different interfacial regions. While the Str. enolase is a homo-octamer, it is composed of a tetramer of dimers in which the orientation of the monomers alternates between which face is up (see Fig 1A). In this Figure, the left subunit and the central subunit form the dimer-dimer interface. The central subunit and the right subunit form the monomer-monomer interface. Fig 1B. The two images are rotated through the x-axis by 180°. The yellow residues are the carboxy-terminus. They are found at the dimer-dimer interface but not at the monomer-monomer. One by one removal of the eight residues destabilizes the octamer and results in dissociation of the octamer into components. (ZIP) [file pone.0206338.s001.zip › S1 Fig.tif]

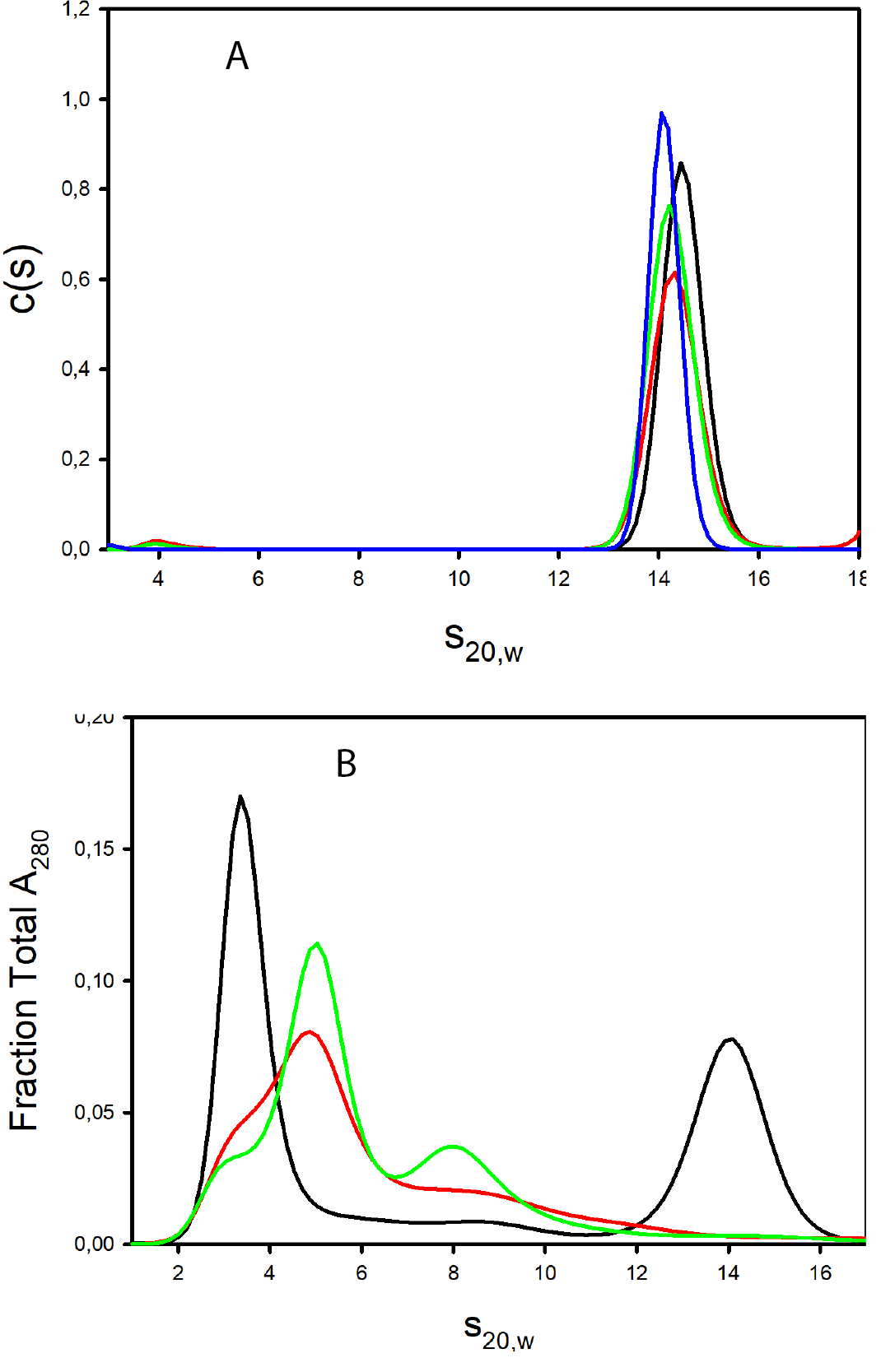

Supplement: S2 Fig — S2A. The influence of different buffers on Str.enolase 137/363 and Str. enolase 137/363–2. Str. enolase 137/363 was incubated in four different buffers and centrifuged in the AUC. The buffers used were: KP1 + Mg (II) (black), KPi (red), KPi + Mg(II) + NaCl (green), and TME-SO4 (blue). The vast majority of the protein migrated as octamers and was not influenced by the buffers used here. S2B. Str. enolase 137/363–2. The black trace represents the protein in TME-SO4. The red trace is the protein in KPi + NaCl; the green trace is in KPi + NaCl + imidazole. The absence of Mg(II) and the presence of NaCl promote dissociation of the native octamer into intermediate species. The peak at s20,w = 3 represent monomers, that at 5.0 corresponds to dimers. The peak at 7.9 is probably tetramers and that at 14 is octamers. (TIF) [file pone.0206338.s002.tif]
