# Supplementary material for: The influence of truncating the carboxy-terminal amino acid residues of streptococcal enolase on its ability to interact with canine plasminogen
Source: PLoS One. 2019 Jan 17;14(1):e0206338. doi: 10.1371/journal.pone.0206338 (PMC6336276; doi:10.1371/journal.pone.0206338)
Supplement: S1 Text — (DOC) [file pone.0206338.s007.doc]

Supplementary text 1.

Protein species running in different AUC cells can be compared directly in DCDT+. Direct comparison without data manipulation is not possible if one wishes to determine if a mixture of protein A and protein B run at something other than the sum of the original fits. The reason for this is as follows. Each DCDT+ determination is the result of choosing a set of scans which is optimal for the particular run. When fitting a sample in which the protein has an s value of 4, we choose one set of the total scans, say scans 75 to 85. When fitting another protein run at the same time with s value 6, a different set of scans would be chosen, say 50 to 61. This gives rise to two sets of fits in which the spacing between the fitted points is different in the two cases. In the third cell of the AUC rotor, we have a mixture of the two proteins. We use DCDT+ to fit solutions to the data. The fit that is returned is good but the data points are spaced differently than in the other two fits because we have started with a different set of scans in each case. We wish to be able to answer the question: Is the sum of fits for pure proteins A and B the same or different from the experimentally determined fit for A+B? We can only answer the question if we can sum the fits for protein A and protein B. We can do this if we fit the individual curves and determine the coefficients of the fits. We then construct a new curve with spacing of the points identical to the actual mixture. We do the same for both individual proteins. This allows us to sum the two curves of the individual proteins. We then compare the sum of the individuals to the experimentally determined mixture.
