# Supplementary material for: The influence of truncating the carboxy-terminal amino acid residues of streptococcal enolase on its ability to interact with canine plasminogen
Source: PLoS One. 2019 Jan 17;14(1):e0206338. doi: 10.1371/journal.pone.0206338 (PMC6336276; doi:10.1371/journal.pone.0206338)
Supplement: S2 Text — (DOC) [file pone.0206338.s008.doc]

Supplementary text 2

time injected

183.31, i proh,0

478.51, hcl,10mM

748.37, ni2so4,100mM

825.19, ni2so4,100mM

3000 Str. enolase 137/363 -4, 2.7 µM

16595.18, dpgn,.036 µM

17188.12, ovalb,30 µM

17422.61, ovalb,30 µM

18441.80, dpgngoog,.036 µM

19034.16, dpgn, 0.11 µM

19613.88, dpgng, 0.11 µM

20198.37, dpgn, 0.33 µM

20784.60, dpgn, 0.33 µM

21479.08, dpgn, 1 µM

22175.10, dpgn, 1 µM

22882.32, dpgn, 3 µM

23566.70, dpgn, 3 µM

24192.09, dpgn, 9.1 µM

24775.91, dpgn, 9.1 µM

25362.90, elution buffer,0

25752.01, edta,10m
